# Supplementary material for: Causal Relationship between Aging and Anorexia Nervosa: A White-Matter-Microstructure-Mediated Mendelian Randomization Analysis
Source: Biomedicines. 2024 Aug 16;12(8):1874. doi: 10.3390/biomedicines12081874 (PMC11351342; doi:10.3390/biomedicines12081874)
Supplement: Supplementary file 1 [file biomedicines-12-01874-s001.zip › Supplementary Figure/Figure S4/Singe SNP plot of MR analysis for TL to ubm-b-1498.pdf]

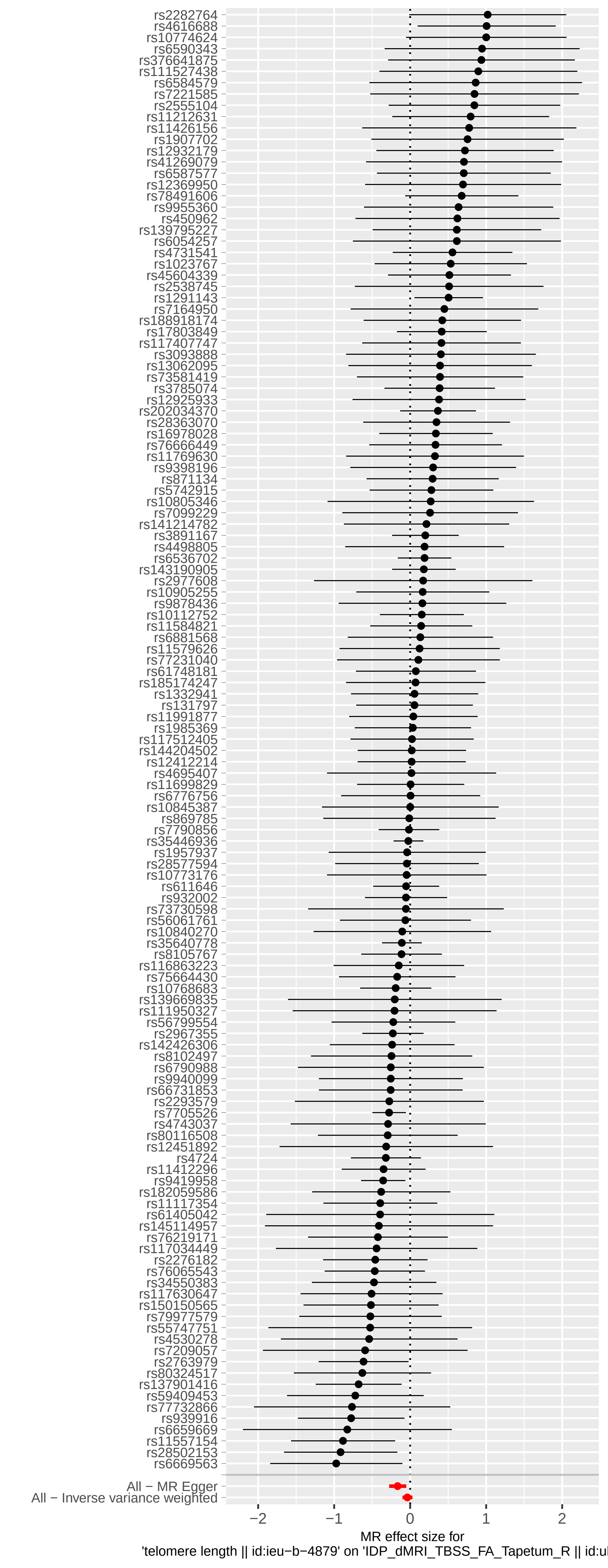
rs2282764
rs4616688
rs10774624
rs6590343
rs376641875
rs111527438
rs6584579
rs7221585
rs2555104
rs11212631
rs11426156
rs1907702
rs12932179
rs41269079
rs6587577
rs12369950
rs78491606
rs9955360
rs450962
rs139795227
rs6054257
rs4731541
rs1023767
rs45604339
rs2538745
rs1291143
rs7164950
rs188918174
rs17803849
rs117407747
rs3093888
rs13062095
rs73581419
rs3785074
rs12925933
rs202034370
rs28363070
rs16978028
rs76666449
rs11769630
rs9398196
rs871134
rs5742915
rs10805346
rs7099229
rs141214782
rs3891167
rs4498805
rs6536702
rs143190905
rs2977608
rs10905255
rs9878436
rs10112752
rs11584821
rs6881568
rs11579626
rs77231040
rs61748181
rs185174247
rs1332941
rs131797
rs11991877
rs1985369
rs117512405
rs144204502
rs12412214
rs4695407
rs11699829
rs6776756
rs10845387
rs869785
rs7790856
rs35446936
rs1957937
rs28577594
rs10773176
rs611646
rs932002
rs73730598
rs56061761
rs10840270
rs35640778
rs8105767
rs116863223
rs75664430
rs10768683
rs139669835
rs111950327
rs56799554
rs2967355
rs142426306
rs8102497
rs6790988
rs9940099
rs66731853
rs2293579
rs7705526
rs4743037
rs80116508
rs12451892
rs4724
rs11412296
rs9419958
rs182059586
rs11117354
rs61405042
rs145114957
rs76219171
rs117034449
rs2276182
rs76065543
rs34550383
rs117630647
rs150150565
rs79977579
rs55747751
rs4530278
rs7209057
rs2763979
rs80324517
rs137901416
rs59409453
rs77732866
rs939916
rs6659669
rs11557154
rs28502153
rs6669563
All − MR Egger
All − Inverse variance weighted
−2
−1
0
1
2
MR effect size for
'telomere length || id:ieu−b−4879' on 'IDP_dMRI_TBSS_FA_Tapetum_R || id:u
